# Machine learning-based model for prediction of clinical deterioration in hospitalized patients by COVID 19

**DOI:** 10.1038/s41598-022-09771-z

**Published:** 2022-05-02

**Authors:** Susana Garcia-Gutiérrez, Cristobal Esteban-Aizpiri, Iratxe Lafuente, Irantzu Barrio, Raul Quiros, Jose Maria Quintana, Ane Uranga, COVID-REDISSEC Working Group, Susana García-Gutiérrez, Iratxe Lafuente, Jose María Quintana, Miren Orive, Nerea Gonzalez, Ane Anton, Ane Villanueva, Cristina Muñoz, Maria Jose Legarreta, Raul Quirós, Pedro Pablo España Yandiola, Mikel Egurrola, Amaia Aramburu, Amaia Artaraz, Leire Chasco, Olaia Bronte, Patricia García, Ana Jodar, Virginia Fernandez, Cristobal Esteban, Naia Mas, Esther Pulido, Itxaso Bengoetxea, Antonio Escobar Martínez, Amaia Bilbao, Iñigo Gorostiza, Iñaki Arriaga, José Joaquín Portu Zapiarain, Naiara Parraza, Milagros Iriberri, Rafael Zalacain, Luis Alberto Ruiz, Leyre Serrano, Adriana Couto, Oier Ateka, Arantza Cano, Maria Olatz Ibarra, Eduardo Millan, Mayte Bacigalupe, Jon Letona, Andoni Arcelay, Iñaki Berraondo, Xavier Castells, Margarita Posso, Lilisbeth Perestelo, Guillermo Perez Acosta, Candelaria Martín Gonzñalez, Maximino Redondo, Maria Padilla, Adolfo Muñoz, Ricardo Saenz de Madariaga

**Affiliations:** 1 Osakidetza Basque Health Service, Research Unit, Galdakao-Usansolo University Hospital, Barrio Labeaga S/N, 48960 Galdakao, Vizcaya Spain; 2 Cambrian Intelligence SLU, Madrid, Spain; 3 Department of Applied Mathematics and Operational Research, University of the Basque Country, Leioa, Spain; 4 Internal Medicine Service, Hospital Costa del Sol, Malaga, Spain; 5 Osakidetza Basque Health Service, Respiratory Service, Galdakao-Usansolo University Hospital, Galdakao, Spain; 6 Kronikgune Institute for Health Services Research, Barakaldo, Spain; 7 Red de Investigación en Servicios Sanitarios y Enfermedades Crónicas (REDISSEC), Zaragoza, Spain; 8 Respiratory Group, Biocruces Bizkaia Health Research Institute, Barakaldo, Spain; 9 Intensive Care Unit, Galdakao-Usansolo Universitary Hospital, Galdakao, Spain; 10 Emergency Department, Galdakao-Usansolo Universitary Hospital, Galdakao, Spain; 11 Hospital at Home, Galdakao-Usansolo Universitary Hospital, Galdakao, Spain; 12 Research Unit, Basurto Universitary Hospital, Bilbao, Spain; 13 Respiratoty Service, Basurto Universitary Hospital, Bilbao, Spain; 14 Internal Medicine, Bioaraba Institute, Vitoria, Spain; 15 Research Unit, Bioaraba Institute, Vitoria, Spain; 16 Internal Medicine, Donostia Universitary Hospital, San Sebastián, Spain; 17 Respiratory Service, Santa Marina Hospital, Bilbao, Spain; 18 Pharmacy Service, Urduliz Hospital, Urduliz, Spain; 19 Central Services Osakidetza, Vitoria, Spain; 20 Department of Health, Basque Government, Vitoria, Spain; 21 Unidad de Calidad e Investigacion, Hospital del Mar, Barcelona, Spain; 22 Servicio de Evaluación y Planificación del Servicio Canario de la Salud, Canary Islands, Tenerife, Spain; 23 Intensive Care Unit, Complejo Hospitalario Universitario Insular-Materno Infantil de Gran Canaria, Gran Canaria, Spain; 24 Internal Medicine, Hospital Universitario de Canarias, Tenerife, Spain; 25 Research Unit, Hospital Costa del Sol, Marbella, Spain; 26 Unidad de Salud Digital ISCIII, Madrid, Spain

**Keywords:** Health care, Risk factors

## Abstract

Despite the publication of great number of tools to aid decisions in COVID-19 patients, there is a lack of good instruments to predict clinical deterioration. COVID19-Osakidetza is a prospective cohort study recruiting COVID-19 patients. We collected information from baseline to discharge on: sociodemographic characteristics, comorbidities and associated medications, vital signs, treatment received and lab test results. Outcome was need for intensive ventilatory support (with at least standard high-flow oxygen face mask with a reservoir bag for at least 6 h and need for more intensive therapy afterwards or Optiflow high-flow nasal cannula or noninvasive or invasive mechanical ventilation) and/or admission to a critical care unit and/or death during hospitalization. We developed a Catboost model summarizing the findings using Shapley Additive Explanations. Performance of the model was assessed using area under the receiver operating characteristic and prediction recall curves (AUROC and AUPRC respectively) and calibrated using the Hosmer–Lemeshow test. Overall, 1568 patients were included in the derivation cohort and 956 in the (external) validation cohort. The percentages of patients who reached the composite endpoint were 23.3% vs 20% respectively. The strongest predictors of clinical deterioration were arterial blood oxygen pressure, followed by age, levels of several markers of inflammation (procalcitonin, LDH, CRP) and alterations in blood count and coagulation. Some medications, namely, ATC AO2 (antiacids) and N05 (neuroleptics) were also among the group of main predictors, together with C03 (diuretics). In the validation set, the CatBoost AUROC was 0.79, AUPRC 0.21 and Hosmer–Lemeshow test statistic 0.36. We present a machine learning-based prediction model with excellent performance properties to implement in EHRs. Our main goal was to predict progression to a score of 5 or higher on the WHO Clinical Progression Scale before patients required mechanical ventilation. Future steps are to externally validate the model in other settings and in a cohort from a different period and to apply the algorithm in clinical practice.

**Registration:** ClinicalTrials.gov Identifier: NCT04463706.

## Introduction

At 15 months after the declaration of the pandemic, there have been more than 160 M confirmed cases of coronavirus disease 2019 (COVID-19) worldwide and nearly 3.5 M in Spain^1^. Though vaccines have been available since December 2020, vaccination rates are uneven across countries. This fact, together with the emergence of new variants of the virus, leads to uncertainty about when global immunity will be achieved^2^. On the other hand, in clinical settings, physicians face patients arriving at hospitals with markedly different characteristics, probably influenced by the age groups vaccinated at each point in time and differences in virulence between strains^3^.

One of the main complications of COVID-19 is acute respiratory distress syndrome (ARDS). Clinical manifestations may be relatively mild in almost all patients, especially in early stages of the disease. These patients might not complain of dyspnea and have no significant increase in respiratory rate or respiratory distress^4^. The challenge for managers and physicians in this context is to identify patients at risk of developing severe forms of the disease with the aim of allocating resources and adequate treatments. Almost all the literature available concerning the prediction of prognosis in COVID-19 has used death^5,6^ or ICU transfer^7,8^ as the main outcome for developing models, with the aim of stratifying patients by risk of poor course. Recently, Gupta et al. have published the 4C Deterioration model for identifying patients at risk of needing ventilatory support^9^.

Our hypothesis is that is possible to predict clinical deterioration in hospitalized patients before the need for intensive ventilatory support. Knowledge about the characteristics of such patients must be the basis of decision support systems to improve triage systems, allowing physicians to decide advanced treatments and, thereby, avoid ICU admission.

## Methods

### Data collection

COVID19-Osakidetza is a sub-study within COVID19-REDISSEC (clinicaltrials.gov # NCT04463706), a prospective cohort study recruiting patients infected by severe acute respiratory syndrome coronavirus 2 (SARS-CoV-2) confirmed by naso- and/or oropharyngeal swab polymerase chain reaction (PCR). We used anonymized patient level data from patients with a confirmed COVID-19 admitted to one of four public hospitals in the Basque Country. Participating hospitals serve a population of approximately 1.2 million and provide tertiary referral services to the surrounding region.

We excluded any patients admitted to these hospitals in the same period but who died in the emergency department or were, immediately or in the first 24 h of the hospital stay, admitted to a critical care unit or reached the endpoint. We also excluded patients who arrived at hospital more than 10 days after their first positive PCR test or tested positive by PCR later than 7 days after admission.

Data were internally stored and managed by the Osakidetza-Basque Public Health System. After anonymisation and removal of protected health information, the data were released in a text-delimited format for research purposes. Patient-level data were collected for the initial analyses in our study. The study protocol was approved by the Ethics Committee of the Basque Country (reference PI2020059). The Basque Country Ethics Committee (PI2020059) approved the waiver of informed consent**.** The study was carried out in accordance with the relevant guidelines and regulations.

We collected data on demographic variables (age, sex), comorbidities and baseline treatments. Comorbidities were assessed based on International Classification of Diseases, Tenth Revision codes that were active on the patients’ electronic health records (EHR) on arrival to the emergency department (ED). We categorized them according to whether the date of diagnosis was in the previous year or earlier. Baseline treatments were assessed based on the Anatomical, Therapeutic and Chemical/Defined Daily Dose (ATC/DDD) index in the EHR, and we categorized them into treatments prescribed in the last 6 months, between > 6 and 12 months earlier and between > 12 months and 5 years earlier (older treatments being excluded). Patients were considered to have no comorbidities or treatments if none were documented in the EHR.

Regarding hospitalization history, we collected information from the ED and up to the first 24 h after admission. This information was related to vital signs: temperature, blood pressure, respiratory and heart rate, oxygen saturation by pulse oximetry and fraction of inspired oxygen, as well as treatments prescribed (ATC/DD). We also gathered data on the most recent lab results (for tests performed up to 1 month before admission).

Our outcome was a composite endpoint defined as need for intensive ventilatory support (with at least standard high-flow oxygen face mask with a reservoir bag for at least 6 h and need for more intensive therapy afterwards or Optiflow high-flow nasal cannula or noninvasive or invasive mechanical ventilation) and/or admission to a critical care unit and/or death during hospitalization.

All the data we used were structured data.

### Datasets

1. *Development and internal validation dataset* We downloaded patient-level data for the period March 1 to April 30, 2020. We randomly split this derivation cohort, without replacement and randomly assuming a uniform distribution, to create two independent sets of patients, assigning 70% of the sample for development of the prediction model and 30% for internal validation. Data were pre-processed to address quality issues, such as allowing the elimination of repeated entries for some patients and exclusion of features with more than 25% of missing data from the list of potential predictor variables.
2. *Validation dataset* Furthermore, a prospective validation set of patients, independent from the other datasets, referred to as the validation dataset, was composed of other patients with COVID-19 admitted from May 1 to October 7, 2020. We used this dataset as the external validation sample and it was pre-processed using the same process as that used the development and internal validation dataset. Further, the demographic and clinical data recorded for these patients were consistent with those of the patients in the development and internal validation cohorts.

In all the resulting datasets, we performed univariate analyses of the differences between patients whose condition did and did not deteriorate using Student’s t test for continuous features and the χ^2^ test for categorical features. We set the p value threshold for significance at 0.05 in these and other analyses in this study.

### Development and validation of the predictive model

We combined the aforementioned information for machine learning analysis using the CatBoost algorithm^10^. Since it is a tree-based model, data normalization is not necessary and categorical variables do not need to be pre-processed.

The contribution of each feature to the model’s prediction was assessed using the Shapley Additive Explanations (SHAP) approach, which ensures high local accuracy, stability against missing data, and consistency in feature impact^11^. The SHAP values were calculated using https://github.com/slundberg/shap. This is a unified approach for explaining the outcome of any machine-learning model. SHAP values evaluate the importance of the output resulting from the inclusion of feature A for all combinations of features other than A. Features being shown in blue indicates that their values are less likely to predict the outcome while values of those shown in red are more likely to be predictive.

We performed four-fold cross-validation in the training set to identify the optimal hyperparameters through a random hyperparameter search and compared the training models through fourfold cross-validation area under the receiver operating curve (AUROC). We also used logistic regression implemented using the Scikit-learn library as baseline model to evaluate and compare.

Different models obtained in the development set were applied to the internal validation set and the model that performed the best based on the AUROC in this latter set was selected. We also calculated the area under precision recall curve (AUPRC) in development, internal validation and external validation sets.

We explored the calibration of models by means of a calibration plot and the Hosmer–Lemeshow Test^12^, which assesses whether or not the observed event rates match expected event rates in subgroups of the model population, identifying subgroups as the deciles of fitted risk values. Based on the distribution of the outcome across the predicted probability in the development and internal validation dataset, we created five risk groups, which were tested in the external validation dataset. We calculated the performance parameters for each risk group.

SAS software was used for the univariate analysis and Python to pre-process data and develop the model with the CatBoost method. Finally, the model was calibrated using R software.

### Ethics approval

The study protocol was approved by the Ethics Committee of the Basque Country (reference PI2020059).

### Consent for publication

The authors give their consent for publication.

## Results

### Data collection

**Figure 1:**
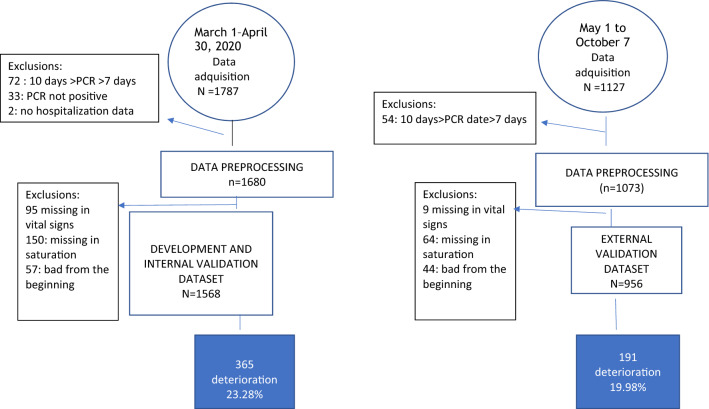
Flow chart.

For the analysis, 1568 patients were included in the derivation set, corresponding to 87% of the PCRs selected, while 956 patients were included in the validation set, corresponding to 85% of the PCRs selected. The percentages of patients who reached the composite endpoint were 23.3% vs 20.0% respectively (p value = 0.05) (Fig. 1).

### Datasets

There were differences between cohorts in sex and age, patients in the external validation dataset being younger (67.42 [Standard Deviation (SD):16] in the derivation dataset vs 65.75 [SD: 20], p = 0.03). There were no differences in comorbidities between cohorts except for dementia (2.36% in derivation vs 4.18 in external validation dataset, % in derivation and validation datasets respectively, p = 0.01). There were more patients taking chronic medications in the external validation dataset. We encountered statistically significant differences in all baseline medications considered except for antihypertensives and immunosuppressants. Regarding vital signs on arrival, no differences were detected except for temperature and oxygen saturation, being lower and higher respectively in the validation sample than in the development sample. We also found differences in several lab results, specifically, inflammation makers, namely, lactate dehydrogenase (LDH) and C-reactive protein (CRP), being lower in the development sample (Table 1). Suplementary Information Table S1 shows more information about differences between cohorts.

**Table 1:** Characteristics of the patients included in derivation and validation cohorts.

Variable	Development and internal validation (n = 1568)	External validation (n = 956)	p-value	Missing
**Sociodemographics**				
Sex (male)			0.01	
Age*	67.42 (16)	65.75 (20)	0.03	
Vital signs*				
Temperature MAX	37.28 (0.92)	37.07 (0.87)	< 0.0001	193/160
SpO2 MIN	94.27 (2.87)	95.18 (2.42)	< 0.0001	815/458
Laboratory test*				
Glucose	122 (44.29)	131.6 (59.17)	< 0.0001	3/12
Urea	45.55 (35)	45 (34.2)	0.60	4/15
Sodium	137.5 (4.27)	138.3 (4.21)	< 0.0001	10/14
Potassium	4.13 (0.51)	4.06 (0.51)	0.0017	46/36
Dimer D	1777 (4793)	1317 (2590)	0.0049	316/153
Prothrombin time	83.7 (21.07)	85.33 (19.64)	0.05	106/23
LDH	308 (127)	277 (269)	< 0.0001	237/170
C-reactive protein	83 (76)	73.6 (69.35)	0.0022	13/27
Procalcitonine	0.37 (2.58)	0.52 (4.12)	0.37	230/189
**Outcome**	365 (23.28)	191 (19.98)	0.05	
VMK 100%	255 (16.26)	79 (8.26)	< 0.0001	
Optiflow	87 (5.55)	73 (7.64)	0.04	
NIMV	45 (2.87)	15 (1.57)	0.0374	
ICU admission	78 (4.97)	54 (5.65)	0.46	
Death	180 (11.48)	110 (11.51)	0.98	

*NSAIDS* non-steroidal anti-inflammatory drugs, *MAX* maximun value, *MIN* minimun value, *SpO2* pulse oximetric saturation, *PaO2* partial arterial oxygen concentration, *ALT* alanine aminotransferase, *LDH* Lactate dehydrogenase, *hs-cTnT* high-sensitivity cardiac troponin T, *RDW* red blood cell distribution width, *VMK100%* standard-high-flow-oxygen-facemask with reservoir-bag at least during 6 h and need for more intensive therapy afterwards, *Optiflow (TM)* high-flow-nasal-cannula, *NIMV* nor invasive mechanical ventilation, *ICU* intensive care unit.

Data are given as frecuencies and percentages except for *, expressed as means and standard deviation.

Table 2 shows the relationship between predictors and the outcome in the development and internal validation dataset and external validation dataset. Among patients who deteriorated, the percentage of males was higher in the derivation dataset (56% vs 69%, p < 0.0001), while there were no differences in the external validation dataset (p = 0.10). Deteriorated patients were older in both datasets. Further, in both datasets, cerebrovascular diseases were more frequent in deteriorated patients, as were chronic pulmonary disease, diabetes, kidney disease and cancer, and in general, they took chronic medications more frequently and had lower oxygen saturation on arrival at the ED. They also presented with higher values of CRP and LDH, lower platelet counts and higher values of mean corpuscular volume (MCV) and red blood cell distribution width (RDW) in both cohorts. Further, in these patients with a poor course, lymphocyte counts were lower in the external validation dataset, while neutrophil counts and D-dimer levels were elevated in both cohorts. Supplementary Information Table S2 shows univariate analysis.

**Table 2:** Univariate analysis, relationship between predictors and outcome in development-internal validation and external validation datasets.

Variable	Missing	Deteroration No (n = 1203)	Deterioration Yes (n = 365)	p-value	Missing	Deteroration No (n = 765)	Deterioration Yes (n = 191)	p-value
**Sociodemographics**								
Sex (male)		681 (56.61)	252 (69.04)	< 0.0001		406 (53.07)	114 (59.7)	0.10
Age*		65.29 (16)	74.53 (14)	< 0.0001		62.94 (19.8)	77 (14.7)	< 0.0001
**Vital signs**								
Temperature MAX	153/40	37.23 (0.9)	37.46 (1)	0.0002	127/33	37.07 (0.83)	37.04 (1.02)	0.75
SpO2 MIN	539/276	94.53 (2.5)	92.3 (4.33)	< 0.0001	322/136	95.35 (2.3)	93.70 (2.62)	< 0.0001
**Laboratory test**								
Glucose	2/1	118.1 (40.33)	134.3 (53.56)	< 0.0001	12/0	128.1 (56.41)	145.5 (67.32)	0.001
Urea	10/4	41 (28)	60.65 (47.33)	< 0.001	3/0	40.21 (27.53)	62.85 (48.89)	< 0.00011/0
Sodium	7/4	137.4 (3.70)	137.5 (5.77)	0.79	2/0	138.3 (3.91)	138.2 (5.24)	0.73
Potassium	33/13	4.11 (0.50)	4.19 (0.55)	0.01	40/6	4.06 (0.49)	4.07 (0.57)	0.70
Dimer D	226/90	1516.2 (3814.6)	2703.2 (7207)	0.0090	133/20	1185.5 (2264.3)	1803.2 (3509.5)	0.03
Prothrombin time	79/27	85 (20.31)	79.33 (23)	< 0.0001	21/2	86.39 (18.94)	81.18 (21.72)	0.0028
LDH	176/61	291 (95.61)	365 (187)	< 0.0001	148/22	269.1 (96.66)	304.6 (130.3)	0.001
C-reactive protein	12/1	71.07	120.6 (87.3)	< 0.0001	15/2	66.90 (65.69)	99.64 (76.93)	< 0.0001
Procalcitonine	182/48	0.30 (2.74)	0.60 (1.95)	0.03	165/24	0.51 (4.56)	0.56 (1.76)	0.81
Red blood cells	3/0	4.63 (0.61)	4.5 (0.68)	0.0017	7/1	4.6 (0.64)	4.42 (0.73)	0.0018
Haemoglobin	3/0	13.74 (1.79)	13.5 (2.02)	0.0467	7/9	13.56 (1.9)	13.11 (2.02)	0.0038
Haematocrit	3/0	42.13 (5.21)	41.79 (6.03)	0.33	7/1	41.21 (5.41)	40.13 (6.03)	0.01
Mean corpuscular volume	3/0	91.26 (5.96)	93.11 (6.69)	< 0.0001	7/1	89.94 (6.30)	91.39 (6.6)	0.0052
RDW	3/0	13.18 (1.60)	13.91 (1.89)	< 0.0001	7/1	13.49 (1.73)	14.18 (2.13)	< 0.0001
Platelets	3/0	201.5 (82.43	171.6 (64.93)	< 0.0001	7/1	206.3 (82.70)	174.3 (72.93)	< 0.0001
Leucocytes	3/0	6.71 (2.88)	7.7 (6)	0.0023	7/0	6.81 (3.43)	7.15 (3.57)	0.24
Limphocytes	6/2	1.18 (0.62)	1.33 (4.81)	< 0.0001	22/6	1.22 (0.65)	0.95 (0.60)	< 0.0001
Neutrophils	6/2	4.92 (2.59)	5.85 (3.33)	< 0.0001	22/6	4.86 (2.85)	5.48 (3.16)	0.0098
Basophils	6/2	0.02 (0.02)	0.02 (0.04)	0.86	22/6	0.02 (0.0006)	0.02 (0.00148)	0.17
Monoytes	6/2	0.54 (0.31)	0.48 (0.47)	0.03	22/6	0.52 (0.28)	0.50 (0.47)	0.67
Eosinophils	6/2	0.04 (0.14)	0.02 (0.04)	< 0.0001	22/6	0.05 (0.27)	0.02 (0.03)	0.0009

*NSAIDS* non-steroidal anti-inflammatory drugs, *MAX* maximum value, *MIN* minimum value, *SpO2* pulse oximetric saturation, *PaO2* partial arterial oxygen concentration, *ALT* alanine aminotransferase, *LDH* Lactate dehydrogenase, *hs-cTnT* high-sensitivity cardiac troponin T, *RDW* red blood cell distribution width, *VMK100%* standard-high-flow-oxygen-facemask with reservoir-bag at least during 6 h and need for more intensive therapy afterwards, *Optiflow (TM)* high-flow-nasal-cannula, *NIMV* nor invasive mechanical ventilation, *ICU* intensive care unit.

### Development and validation of the predictive model

**Figure 2:**
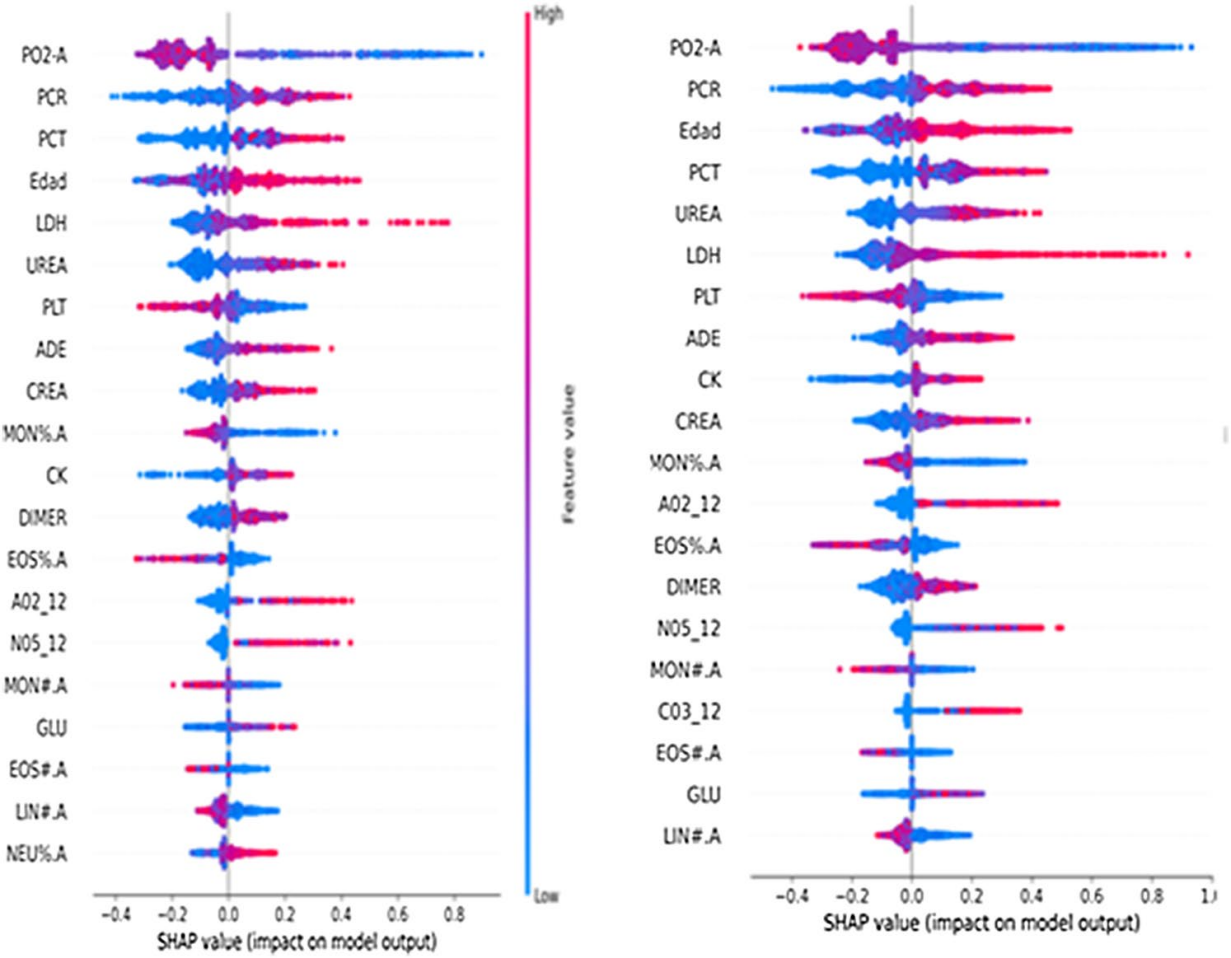
Main predictors in catboost model in (**a**) derivation-internal validation and (**b**) external validation datasets. *PO2-A* partial arterial oxygen concentration, *PCR* C-reactive proteine, *PCT* procalcitonine, *Edad* age, *LDH* lactate dehydrogenase, *PLT* platelets, *ADE* RED blood cell distribution width, *CREA* creatinine, *Mon%A* Total count of monocytes, *CK* creatine kinase, *Dimer* D dimer, *EOS%A* Percentage of eoshinophils, *AO2_12* Antacids in the last 12 months, *N05_12* neuroleptics in the last 12 months, *MON#A* total count of monocytes, *GLU* glucose, *EOS#A* TOTAL count of eosynophils, *lin#A* total count of lynphocites, *Neu%A* percentage of neutrophils, *CO3_12* diuretics in the last 12 months.

**Figure 3:**
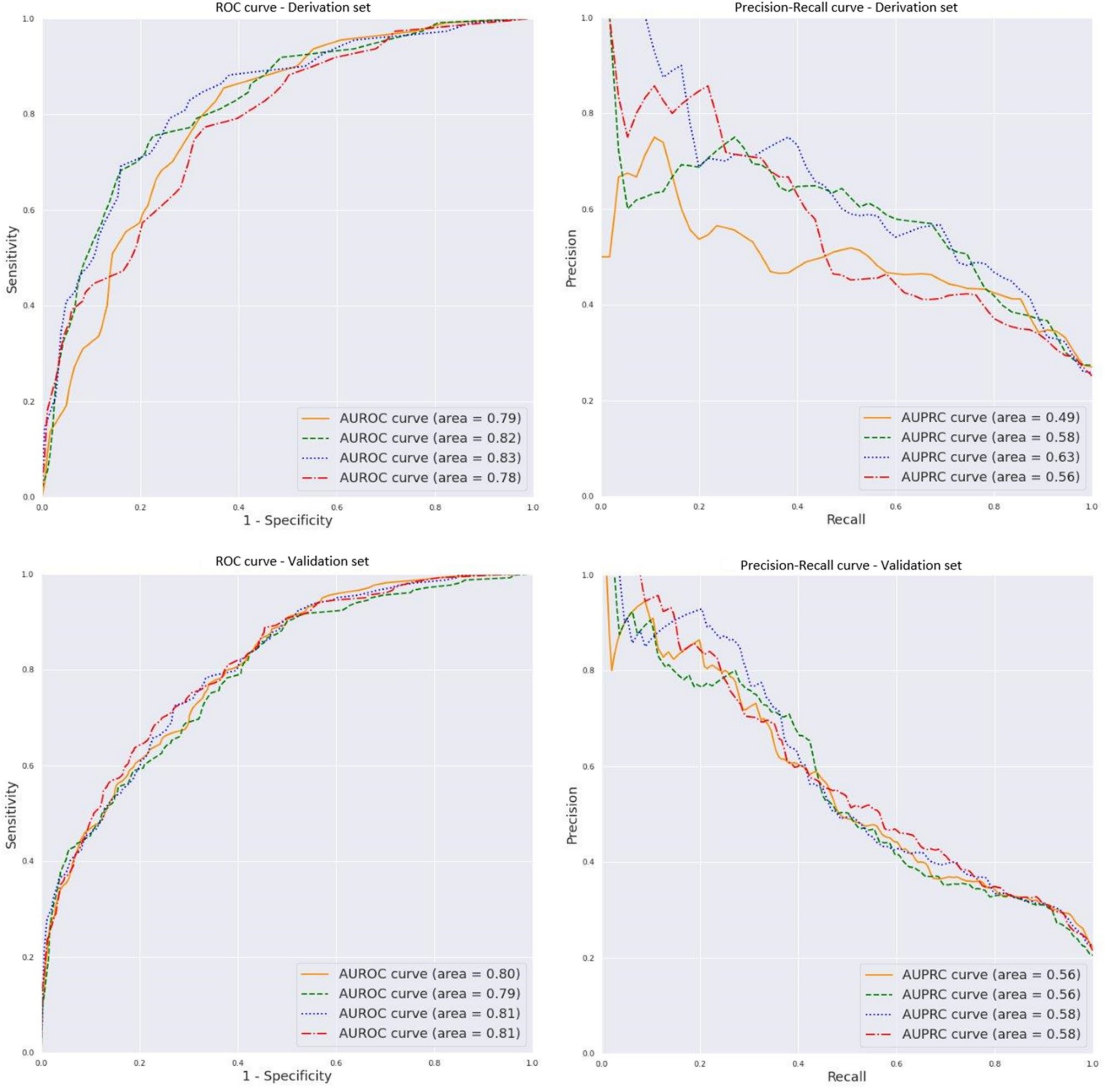
Predictive performance of the catboost model in (**a**) derivation and (**b**) validation sets.

Figure 2 presents main variables in the CatBoost model. The strongest predictors of clinical deterioration were arterial blood oxygen pressure, followed by age, levels of several markers of inflammation (procalcitonin, LDH, CRP) and alterations in blood count and coagulation. Some medications, namely, ATC AO2 (antiacids) and N05 (neuroleptics) were also among this group of main predictors, together with C03 (diuretics). The predictive performance of the CatBoost model in the derivation and validation sets are shown in Fig. 3.

We used SHAP values to provide consistent and locally accurate attribution values for each feature within the prediction model. Patients who reached the endpoint obtained the lowest values for partial pressure of oxygen, platelets, lymphocytes, monocytes and eosinophils and the highest values for CRP, age, procalcitonin, urea, LDH, RDW, creatine kinase, creatinine, D-dimer and glucose. In the case of baseline treatments, which were dichotomous variables, those who took neuroleptics, antacids and/or diuretics were more likely to deteriorate than those who did not.

**Figure 4:**
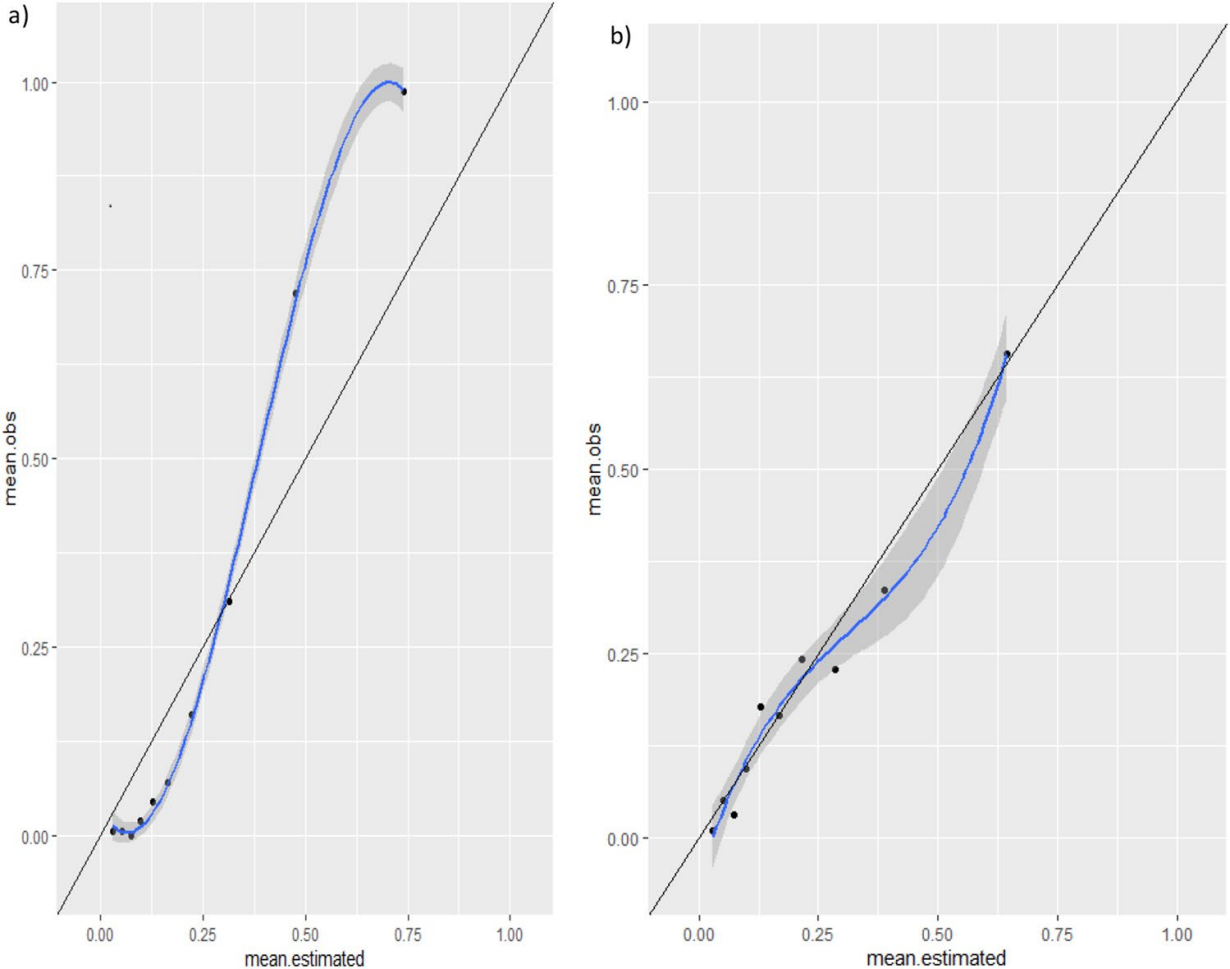
Calibration performance in (**a**) derivation and (**b**) validation sets.

Calibration plots are shown in Fig. 4, the Hosmer–Lemeshow test p value being < 0.001 in the development cohort and 0.3654 in the validation cohort.

We explored the distribution of the outcome across the predicted probability based on the scores from the CatBoost classifier. We considered five different cut-off points (> 0.04, > 0.13, > 0.23, > 0.47 and > 0.82) to classify patients as reaching or not reaching the endpoint based on predicted values of probability. Table 3 shows the performance parameters for each cut-off point, the lowest group being the most sensitive and the highest group the most specific. The negative predictive value ranged between 0.81 and 0.99 across the risk groups. In addition, the variable representing patients risk stratification was defined considering the cut-off points mentioned above and creating complementary risk groups.

**Table 3:** Sensitivity, specificity, and positive and negative predictive values according to different cutoff points in both, (a) derivation-internal validation and (b) external validation datasets.

Risk groups	No patients	Derivation cohort				No (%) deteriorated in complementary risk groups
		Sensitivity	Specificity	Positive predictive value	Negative predictive value	
Score > 0.04	1424	1	0.12	0.26	0.99	1 (0.69%)
Score > 0.13	851	0.98	0.59	0.42	0.99	9 (1.26%)
Score > 0.23	530	0.90	0.83	0.62	0.96	38 (3.66%)
Score > 0.47	227	0.59	0.99	0.94	0.89	151 (11.26%)
Score > 0.82	31	0.08	1	1	0.78	334 (21.73%)

Risk groups	No patients	Validation cohort				No (%) deteriorated in complementary risk groups
		Sensitivity	Specificity	Positive predictive value	Negative predictive value	
Score > 0.04	862	0.99	0.12	0.22	0.99	1 (1.06%)
Score > 0.13	525	0.86	0.53	0.31	0.94	26 (6.03%)
Score > 0.23	313	0.65	0.75	0.40	0.90	67 (10.42%)
Score > 0.47	98	0.33	0.95	0.64	0.85	128 (14.92%)
Score > 0.82	13	0.06	1	0.92	0.81	179 (18.98%)

Risk cut-off values were defined by the total point score for an individual, which represented low (< 2% mortality rate), intermediate (2–14.9%), or high risk (≥ 15%) groups, similar to commonly used pneumonia risk stratification scores.

## Discussion

We have developed and validated a clinical prediction model including routinely available information recorded in the EHR. This model includes chronic treatments prescribed at baseline, lab results and vital signs on arrival at hospital and predicts poor course in patients with COVID-19 before transfer to an ICU. Therefore, it is expected that it could improve triage systems allowing earlier identification of patients who are likely to need intensive ventilatory support. These decision support systems must be feasible, and have to be continuously updated to detect variations in patients’ characteristics that make them differently vulnerable.

Deciding whether or not to intubate is a critical aspect of caring for patients seriously ill with COVID-19^13^. We excluded patients in which emergent intubation was required, and hence, the algorithm is designed to detect poor prognosis in patients who arrive at a hospital ward with severe COVID, requiring oxygen therapy. We believe ICU transfer is not a good outcome to consider if seeking to identify all patients who have a poor course in COVID-19 as it results in an under diagnosis of severe forms of the disease. We believe that the outcome should be a composite endpoint of ICU transfer, mortality, and need for intensive ventilatory support, corresponding to a score of 5 or more on the WHO Clinical Progression Scale^14^, including patients who will need standard high-flow oxygen face mask with a reservoir bag, if we want to identify the whole spectrum of patients who develop severe forms of this disease.

Izquierdo et al. have recently published a predictive model of ICU admission of patients with COVID-19 based on a sample of 10,504 patients in a region of Spain. One of the limitations of that model is that they did not include lab results or baseline treatments in their prediction model, their conclusion being that age, fever, and tachypnea were the main predictors of ICU admission^8^. We believe that these predictors occurred late in the presentation of the disease; in contrast, our work provides clinicians and health managers an aid to take earlier decisions.

The recently published 4C Deterioration model used a composite primary outcome of in-hospital clinical deterioration considering initiation of ventilatory support from the start of noninvasive ventilation (score of 6 on the WHO Clinical Progression Scale). We defined intensive ventilation as the need for 100% O2, including patients not candidates for mechanical ventilation but whose condition deteriorated and this is a main strength of our work. We included all data available for each patient related to the information we wanted to enter into the model: comorbidities, treatments, lab results and vital signs, without a priori definitions for each variable. The goal of Gupta et al. in proposing the 4C deterioration model was that it should be easily usable^9^. Our main goal was, by contrast, to achieve the greatest possible accuracy in our predictions, since we envisaged its implementation in the EHR, and depending on the pre-selected decision threshold, physicians would receive the performance parameters automatically to aid their decision-making.

We derived the model in the first months of pandemic, and therefore, differences between patients in the derivation and external validation cohorts were expected. While there were differences in sociodemographic characteristics, we did not detect differences in comorbidities except for dementia. This could be due to the availability of hospital beds beyond the first months of the pandemic, since in the first months, patients with dementia were more likely to be treated in care homes. Other differences were encountered in inflammatory markers and other lab results, values being slightly better in patients in the validation cohort than in those in the derivation cohort; nevertheless, these differences were not clinically significant.

In any case, our model was sufficiently robust to identify patients at risk of clinical deterioration under new conditions, presenting excellent performance properties in other settings/circumstances.

In the literature, there is at least moderate evidence of predictive value in COVID-19 for almost all of our main predictors (partial pressure of oxygen, age, PCT, LDH, CRP, BUN, PLT, CK, D-dimer, creatinine, and lymphocyte count)^15^. RDW on admission and an increase therein during hospitalization have been proposed as predictors of mortality, especially in younger patients^16^. We also found low levels of monocytes and eosinophils to be related to poor course^17^. Such variations in blood cell counts with the progression of the disease are to be expected, but we provide the probability of poor outcomes depending of the magnitude of these variations, adjusted for other characteristics of the patients and this is another strength of our work.

A recent meta-analysis indicated that patients with upper gastrointestinal diseases taking omeprazole may be more vulnerable to COVID-19, without confirmation of severe disease being associated with other drugs in the same ATC group^18^. Some authors have hypothesized that psychiatric patients could be protected against COVID-19 due to being on neuroleptics, but this has not been confirmed in our study^19^. Another possible explanation for antacids and neuroleptics being among the main predictors is that they were acting as a proxy for the overall number of drugs taken by patients. We assessed the number of drugs prescribed among the group of patients who took antacids or neuroleptics compared to that in patients who did not take these types of medications, and we found significant differences. Specifically, patients who took antacids or neuroleptics took eight drugs compared to two in the groups of patients who did not take them (data not shown). Oddy et al. identified diuretics as protectors of ICU admission and cardiac arrest as well being associated with lower oxygen requirements^20^, while Cabezon et al. did not find a poorer prognosis in patients who took diuretics^21^.

Our model presented excellent AUROCs and this indicates that the algorithm is sufficiently sensitive to identify patients at risk of needing intensive ventilatory support and acceptable AUPRC indicates that the algorithm is able to distinguish false positives. We proposed several cut-off points to aid decisions about allocation of care to patients. Another advantage of our model is that it takes into account all the comorbidities and baseline medications recorded, in a more specific way than models for predicting poor prognosis described to date^9,22,23^. Indeed, our machine-learning based model is designed to detect which patients in the pulmonary phase at admission are going to need ventilatory support during their hospital stay. Bardley et al. recommended focusing on features of respiratory compromise rather than circulatory collapse as almost all the predictive models do^23^.

**Figure 5:**
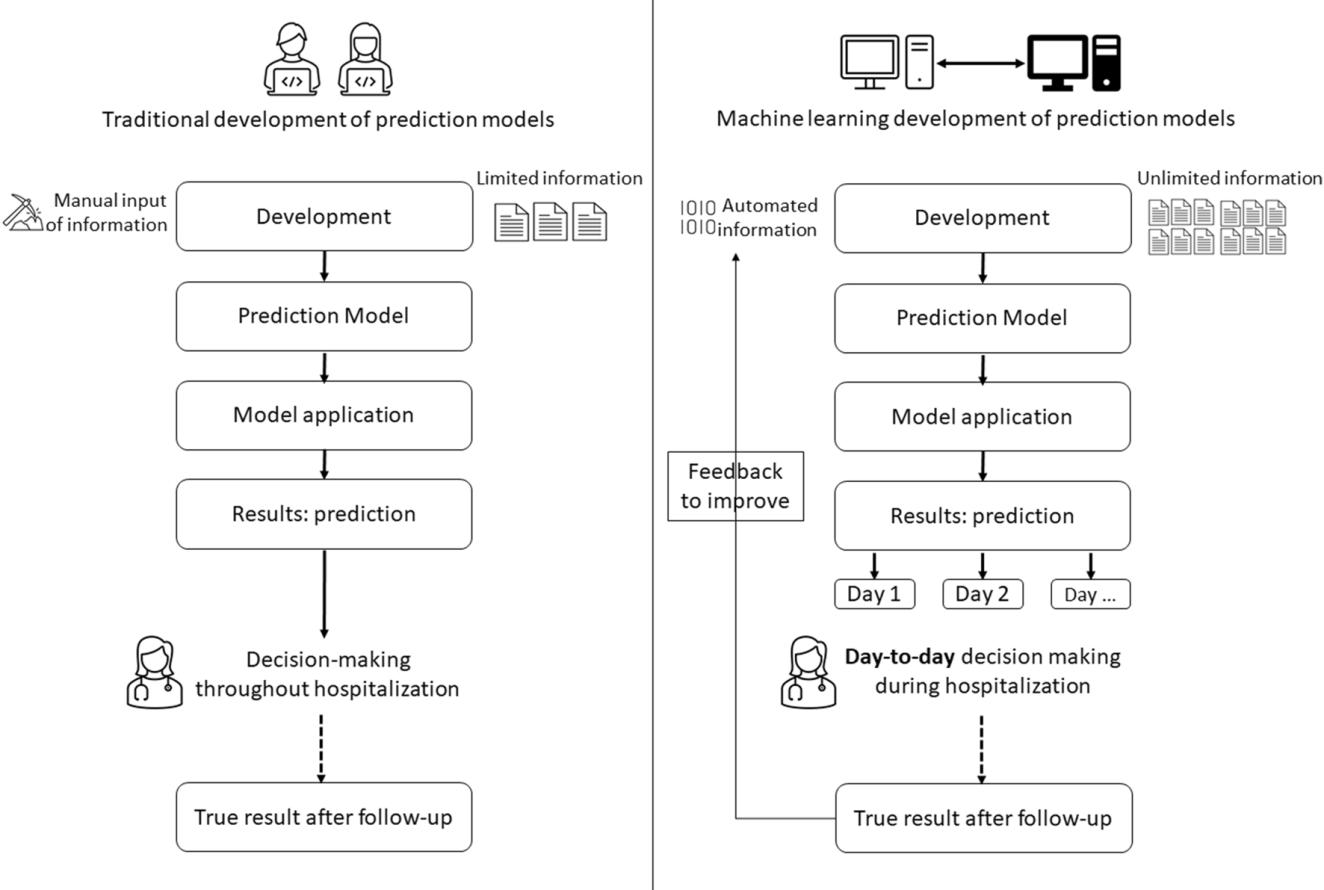
Differences between traditional development and machine-learning based prediction models.

A limitation of our work is the calibration performance of the model in the derivation dataset. Specifically, there is some under prediction when the observed probability of experiencing deterioration is greater than 0.5, while there is a slight over prediction when the observed probability is below 0.25. Nevertheless, the estimated and observed probabilities in the external validation dataset are very well calibrated, which indicates that the model has a good fit when applied to an external sample, although it is true that, as might be expected, the estimates in the 10 groups do not show a monotonic growth. This could be due to differences in the profile of patients across the waves of the pandemic as well as in the response of the health system and medical knowledge of the disease^3^. This leads us to recommend the use of dynamic models, that is, models should be continuously updated after their implementation in clinical practice to adjust them for these differences. Figure 5 outlines differences between traditional models and those based on machine-learning systems. Clinicians are provided with the result of the model automatically, and the model updates with the change in profiles of patients or changes in health system practices, such as changes in treatment protocols. Preprocessing code plus model training will be available on specific request in clinical trials.gov.

## Conclusions

In conclusion, we present a machine-learning based prediction model with excellent performance properties to be implemented in EHRs. Our main goal was to predict progression to a score of 5 or higher on the WHO Clinical Progression Scale prior to patients requiring mechanical ventilation. Future steps are to externally validate the model in other settings and in a cohort from a different time period and to apply the algorithm in clinical practice^14^.

## Supplementary Information

Supplementary Tables.

## Data Availability

Data available on request from the authors.
