# Supplementary material for: Machine learning-based model for prediction of clinical deterioration in hospitalized patients by COVID 19
Source: Sci Rep. 2022 May 2;12:7097. doi: 10.1038/s41598-022-09771-z (PMC9059444; doi:10.1038/s41598-022-09771-z)
Supplement: Supplementary file 1 — Supplementary Tables. [file 41598_2022_9771_MOESM1_ESM.docx]

**SUPPLEMENTARY INFORMATION**

**Supplementary Table S1: Characterstics of the patients included in derivation and validation cohorts**

|  |  |  |  |  |  |
| --- | --- | --- | --- | --- | --- |
| Variable |  | Development and internal validation (n=1568) | External validation  (n=956) | p-value | missing |
| ***Sociodemographics*** |  |  |  |  |  |
| Sex (male) |  |  |  | 0.01 |  |
| Male |  | 933(59.50) | 520(54.39) |  |  |
| Female |  | 635(40.50) | 436(45.61) |  |  |
| Age^*^ |  | 67.42(16) | 65.75(20) | 0.03 |  |
| ***Comorbidities*** |  |  |  |  |  |
| Myocardial infarction |  | 30(1.91) | 16(1.67) | 0.66 |  |
| Congestive heart failure |  | 22(1.28) | 19(2) | 0.26 |  |
| Peripheral vascular disease |  | 74(4.72) | 37(3.87) | 0.31 |  |
| Cerebrovascular disease |  | 89 (5.68) | 63 (6.59) | 0.35 |  |
| Dementia |  | 37(2.36) | 40(4.18) | 0.01 |  |
| Chronic pulmonary disease |  | 181(11.54) | 110(11.51) | 0.98 |  |
| Rheumatic disease |  | 22 (1.40) | 13(1.36) | 0.93 |  |
| Liver disease (mild) |  | 42(2.68) | 21(2.20) | 0.45 |  |
| Diabetes without chronic complication |  | 142(9.06) | 98(10.25) | 0.32 |  |
| Diabetes with chronic complication |  | 39(2.49) | 25(2.62)) | 0.84 |  |
| Renal disease |  | 149(9.50) | 83(8.68) | 0.49 |  |
| Neoplasia |  | 200(12.76) | 101 (10.56) | 0.1 |  |
| Metastatic solid tumor |  | 18(1.15) | 12(1.26) | 0.81 |  |
| ***Basal treatments*** |  |  |  |  |  |
| Antidiabetics |  | 211(13.46) | 198(20.71) | <0.0001 |  |
| Antiacids |  | 274 (17.47) | 246 (25.73) | <0.0001 |  |
| Antihipertensives |  | 24(1.53) | 25(2.62) | 0.05 |  |
| Diuretics |  | 195(12.44) | 212(22.18) | >0.0001 |  |
| Beta-blockers |  | 133(8.48) | 113(11.8) | 0.0061 |  |
| Calciummm channel blockers |  | 129(8.23) | 120(12.55) | 0.0004 |  |
| Renin angiotensin aldosterone system inhibitors |  | 362(23.09) | 278(29.1) | 0.0008 |  |
| Lipid loweing drugs |  | 235(14.99) | 209(20.86) | <0.0001 |  |
| NSAIDs |  | 718(45.8) | 597(62.45) | <0.0001 |  |
| Anticoagulants |  | 374(23.85) | 326 (34.10) | <0.0001 |  |
| Inmunosupressants |  | 29(1.85) | 20(2.1) | 0.67 |  |
| Chronic sistemic steroids |  | 373(23.79) | 310(32.43) | <0.0001 |  |
| Neuroleptics |  | 259 (16.52) | 233(24.37) | <0.0001 |  |
| ***Vital signs **** |  |  |  |  |  |
| Temperature MAX |  | 37.28 (0.92) | 37.07(0.87) | <0.0001 | 193/160 |
| Systolic Blood pressure MIN |  | 124(19.18)) | 125.6(21.36) | 0.08 | 227/157 |
| Systolic Blood pressure MAX |  | 132.3(19.69) | 132 (22.03) | 0.60 | 227/157 |
| Dyastolic blood pressure MIN |  | 72.20(10.72) | 73.12(11.73) | 0.07 | 227/157 |
| Dyastolic blood pressure MAX |  | 77.54(10.85) | 77(11.56) | 0.22 | 227/157 |
| Cardiac rate MIN |  | 75.80(13.63) | 77(15) | 0.07 | 220/152 |
| Cardiac rate MAX |  | 83.75(14.83)) | 82.85(15.77) | 0.18 | 220/152 |
| SpO2 MIN |  | 94.27 (2.87) | 95.18 (2.42) | <0.0001 | 815/458 |
| ***Laboratory test**** |  |  |  |  |  |
| PaO2 |  | 74.61(21.60) | 73.85 (23.86) | 0.51 | 457/376 |
| Glucose |  | 122(44.29) | 131.6(59.17) | <0.0001 | 3/12 |
| Urea |  | 45.55(35) | 45(34.2) | 0.60 | 4/15 |
| Creatinine |  | 1.06(0.72) | 1.08(0.87) | 0.62 | 4/10 |
| ALT |  | 34.84(32.9) | 35.26(36.71) | 0.78 | 15/81 |
| Creatine kinase |  | 163(322) | 193(635) | 0.29 | 712/346 |
| Sodium |  | 137.5(4.27) | 138.3(4.21) | <0.0001 | 10/14 |
| Potassium |  | 4.13(0.51) | 4.06(0.51) | 0.0017 | 46/36 |
| Dimer D |  | 1777(4793) | 1317(2590) | 0.0049 | 316/153 |
| Prothrombin time |  | 83.7(21.07) | 85.33(19.64) | 0.05 | 106/23 |
| Ferritin |  | 754(919) | 715(838.5) | 0.49 | 1155/329 |
| Fibrinogen |  | 601(141) | 584.2(163) | 0.1 | 1067/532 |
| LDH |  | 308(127) | 277(269) | <0.0001 | 237/170 |
| C-reactive protein |  | 83(76) | 73.6(69.35) | 0.0022 | 13/27 |
| Procalcitonine |  | 0.37(2.58) | 0.52(4.12) | 0.37 | 230/189 |
| hs-cTnT |  | 28(51) | 19(21) | 0.001 | 1128/877 |
| Red blood cells |  | 4.60(0.63) | 4.46(0.66) | 0.12 | 3/8 |
| Haemoglobin |  | 13.7(1.85) | 13.47(1.93) | 0.0044 | 3/7 |
| Haematocrit |  | 42(5.4) | 41(5.55) | <0.0001 | 3/8 |
| Mean corpuscular volume |  | 91.7(6.2) | 90.22(6.39) | <0.0001 | 3/8 |
| RDW |  | 13.35(1.7) | 13.63(1.84) | 0.0002 | 3/6 |
| Platelets |  | 194.5(79.7) | 200(82) | 0.10 | 3/7 |
| Leukocytes |  | 6.94 | 6.89(3.5) | 0.72 | 3/7 |
| Lymphocytes |  | 1.21(2.38) | 1.17(0.65) | 0.46 | 8/28 |
| Neutrophils |  | 5.14(2.80) | 5(3) | 0.19 | 8/28 |
| Basophils |  | 0.02(0.03) | 0.02(0.02) | 0.59 | 8/28 |
| Monoytes |  | 0.52(0.35) | 0.52(0.33) | 0.78 | 8/28 |
| Eosinophils |  | 365(23.3) | 191(20) | 0.05 | 8/28 |
| ***Outcome*** |  | 365 (23.28) | 191(19.98) | 0.05 |  |
| VMK 100% |  | 255(16.26) | 79(8.26) | <0.0001 |  |
| Optiflow |  | 87(5.55) | 73(7.64) | 0.04 |  |
| NIMV |  | 45(2.87) | 15(1.57) | 0.0374 |  |
| ICU admission |  | 78(4.97) | 54(5.65) | 0.46 |  |
| Death |  | 180(11.48) | 110(11.51) | 0.98 |  |

NSAIDS: non-steroidal anti-inflammatory drugs; MAX: maximun value; MIN: minimun value; SpO2: pulse oximetric saturation; PaO2: partial arterial oxygen concentration; ALT: alanine aminotransferase; LDH: Lactate dehydrogenase; hs-cTnT: high-sensitivity cardiac troponin T ; RDW: Red blood cell distribution width; VMK100%: standard-high-flow-oxygen-facemask with reservoir-bag at least during six hours and need for more intensive therapy afterwards; Optiflow (TM)-high-flow-nasal-cannula; NIMV: nor invasive mechanical ventilation; ICU : intensive care unit.

Data are given as frecuencies and percentages except for *, expressed as means and standard deviation.

**Supplementary Table S2: Univariate analisis, relationship between predictors and outcome in development-internal validation and external validation datasets**

|  | DEVELOPMENT DATASET | | | | EXTERNAL VALIDATION DATASET | | | |
| --- | --- | --- | --- | --- | --- | --- | --- | --- |
| Variable | Missing | Deteroration  no (n=1203) | Deterioration  Yes (n=365) | p-value | Missing | Deteroration  no (n=765) | Deterioration  yes (n=191) | p-value |
| ***Sociodemographics*** |  |  |  |  |  |  |  |  |
| Sex (male) |  | 681(56.61) | 252(69.04) | <0.0001 |  | 406(53.07) | 114(59.7) | 0.10 |
| Age^*^ |  | 65.29(16) | 74.53(14) | <0.0001 |  | 62.94(19.8) | 77(14.7) | <0.0001 |
| ***Comorbidities*** |  |  |  |  |  |  |  |  |
| Myocardial infarction |  | 24(2) | 6(1.64) | 0.67 |  | 11(1.44) | 5(2.62) | 0.25 |
| Congestive heart failure |  | 14(1.16) | 6(1.64) | 0.47 |  | 14(1.83) | 5(2.62) | 0.48 |
| Peripheral vascular disease |  | 46(3.82) | 28(7.67) | 0.0024 |  | 29(3.79) | 8(4.19) | 0.8 |
| Cerebrovascular disease |  | 55(4.57) | 34(9.32) | 0.0006 |  | 42(5.49) | 21(11) | 0.006 |
| Dementia |  | 25(2.08) | 12(3.29) | 0.18 |  | 27(3.53) | 13(6.81) | 0.04 |
| Chronic pulmonary disease |  | 114(9.48) | 67(18.36) | <0.0001 |  | 80(10.46) | 30(15.71) | 0.04 |
| Rheumatic disease |  | 18(1.50) | 4(1.10) | 0.57 |  | 11(1.44) | 2(1.05) | 0.67 |
| Liver disease (mild) |  | 31(2.58) | 11(3.01) | 0.65 |  | 15(1.96) | 6(3.14) | 0.32 |
| Diabetes without chronic complication |  | 87(7.23) | 55(15.07) | <0.0001 |  | 69(9.02) | 29(15.18) | 0.01 |
| Diabetes with chronic complication |  | 27(2.24) | 12(3.29) | 0.26 |  | 17(2.22) | 8(4.19) | 0.13 |
| Renal disease |  | 85(7.07) | 64(17.53) | <0.0001 |  | 57(7.45) | 26(13.61) | 0.007 |
| Neoplasia |  | 131(10.9) | 69(18.9) | <0.0001 |  | 69(9.02) | 32(16.75) | 0.0019 |
| Metastatic solid tumor |  | 11(0.91) | 7(1.92) | 0.11 |  | 9(1.18) | 3(1.57) |  |
| ***Basal treatments*** |  |  |  |  |  |  |  |  |
| Antidiabetics |  | 134(11.14) | 77(21.10) | <0.0001 |  | 131(17.12) | 67(35.08) | <0.0001 |
| Antiacids |  | 167(13.88) | 107(29.32) | <0.0001 |  | 158 (20.65) | 88(46.07) | <0.0001 |
| Antihipertensives |  | 15(1.25) | 9(2.47) | 0.09 |  | 13(1.70) | 12(6.28) | 0.0004 |
| Diuretics |  | 112(9.31) | 83(22.74) | <0.0001 |  | 143(18.69) | 69(36.13) | <0.0001 |
| Cardiovascular |  | 83(6.90) | 32(8.77) | 0.23 |  | 50(6.54) | 18(9.42) | 0.16 |
| Beta-blockers |  | 84(7) | 49(13.42) | 0.0001 |  | 79(10.33) | 34(17.80) | 0.0042 |
| Calciummm channel blockers |  | 83(7) | 46(12.60) | 0.0005 |  | 79(10.33) | 41(21.47) | <0.0001 |
| Renin-angiotensin aldosterone system inhibitors |  | 244(21) | 118(32.33) | <0.0001 |  | 184(24.05) | 94(49.21) | <0.0001 |
| Lipid loweing drugs |  | 155(13) | 80(22) | <0.0001 |  | 128(16.73) | 81(42.41) | <0.0001 |
| NSAIDs |  | 570(47.38) | 148(40.55) | 0.02 |  | 477(62.35) | 120(62.83) | 0.90 |
| Anticoagulants |  | 254(21) | 120(33) | <0.0001 |  | 224(29.28) | 102(53.40) | <0.0001 |
| Inmunosupressants |  | 23(2) | 6(1.64) | 0.74 |  | 17(2.22) | 3(1.57) | 0.57 |
| Chronic sistemic steroids |  | 270(22.44) | 103 (28) | 0.02 |  | 241(31.50) | 69(36.13) | 0.22 |
| Neuroleptics |  | 164(13.63) | 95(26.03) | <0.0001 |  | 150 (19.61) | 83(43.46) | <0.0001 |
| ***Vital signs*** |  |  |  |  |  |  |  |  |
| Temperature MAX | 153/40 | 37.23(0.9) | 37.46(1) | 0.0002 | 127/33 | 37.07(0.83) | 37.04(1.02) | 0.75 |
| Systolic Blood pressure MIN | 176/51 | 123.3(18.8) | 126.3(20.26) | 0.01 | 127/30 | 125.7(21.72) | 124.9(19.84) | 0.67 |
| Systolic Blood pressure MAX | 176/51 | 131.4(19.2 | 135(21) | 0.005 | 127/30 | 131.6(22.22) | 132.5(21.35) | 0.62 |
| Dyastolic blood pressure MIN | 176/51 | 72.38(1.48) | 71.61(11.46) | 0.28 | 127/30 | 73.69(12.06) | 70.84(10.03) | 0.0022 |
| Dyastolic blood pressure MAX | 176/51 | 77.57(10.37) | 77.44(12.27) | 0.86 | 127/30 | 77.25(11.86) | 75.59(10.19) | 0.07 |
| Cardiac rate Min | 177/43 | 75.2(12.7) | 77.75(16.14) | 0.0096 | 123/29 | 76.77(12.69) | 77.78(16.04) | 0.47 |
| Cardiac rate Max | 177/43 | 83.07(14.3) | 85.92(16.24) | 0.005 | 123/29 | 82.42(15.22) | 84.55(17.74) | 0.16 |
| SpO2 Min | 539/276 | 94.53(2.5) | 92.3(4.33) | <0.0001 | 322/136 | 95.35(2.3) | 93.70(2.62) | <0.0001 |
| ***Laboratory test*** |  |  |  |  |  |  |  |  |
| Glucose | 2/1 | 118.1(40.33) | 134.3(53.56) | <0.0001 | 12/0 | 128.1(56.41) | 145.5(67.32) | 0.001 |
| Urea | 10/4 | 41(28) | 60.65(47.33) | <0.001 | 3/0 | 40.21(27.53) | 62.85(48.89) | <0.00011/0 |
| Creatinine | 3/1 | 1(0.65) | 1.27(0.85) | <0.0001 | 1/0 | 0.97(0.50) | 1.48(1.59) | <0.0001 |
| ALT | 82/23 | 34.8(32) | 35(35.5) | 0.89 | 67/14 | 36.13(38.73) | 31.81(27.17) | 0.08 |
| Creatine kinase | 526/186 | 157.6(346) | 183(208) | 0.22 | 274/72 | 182.9(655) | 233.1(545) | 0.39 |
| Sodium | 7/4 | 137.4(3.70) | 137.5(5.77) | 0.79 | 2/0 | 138.3(3.91) | 138.2 (5.24) | 0.73 |
| Potassium | 33/13 | 4.11(0.50) | 4.19(0.55) | 0.01 | 40/6 | 4.06(0.49) | 4.07(0.57) | 0.70 |
| Dimer D | 226/90 | 1516.2(3814.6) | 2703.2(7207) | 0.0090 | 133/20 | 1185.5(2264.3) | 1803.2(3509.5) | 0.03 |
| PaO2 | 427/70 | 76.89(21.55) | 68.62(20.57) | <0.0001 | 329/47 | 73.9 (23.7) | 73.73(24.5) | 0.94 |
| Prothrombin time | 79/27 | 85(20.31) | 79.33(23) | <0.0001 | 21/2 | 86.39(18.94) | 81.18(21.72) | 0.0028 |
| Ferritin | 884/271 | 743.5 (957) | 789.5(781.4) | 0.63 | 263/66 | 696.4(826) | 790(887) | 0.26 |
| Fibrinogen | 801/266 | 597.1(139) | 617(147.5) | 0.21 | 418/114 | 582.8(165.5) | 591(151.2) | 0.70 |
| LDH | 176/61 | 291(95.61) | 365(187) | <0.0001 | 148/22 | 269.1(96.66) | 304.6(130.3) | 0.001 |
| C-reactive protein | 12/1 | 71.07 | 120.6(87.3) | <0.0001 | 15/2 | 66.90(65.69) | 99.64(76.93) | <0.0001 |
| Procalcitonine | 182/48 | 0.30(2.74) | 0.60(1.95) | 0.03 | 165/24 | 0.51(4.56) | 0.56(1.76) | 0.81 |
| hs-cTnT | 863/265 | 24.61(50.61) | 39.23(5.24) | 0.001 | 701/176 | 15.59(11.71) | 34.67(38.69) | 0.07 |
| Red blood cells | 3/0 | 4.63(0.61) | 4.5(0.68) | 0.0017 | 7/1 | 4.6(0.64) | 4.42(0.73) | 0.0018 |
| Haemoglobin | 3/0 | 13.74(1.79) | 13.5(2.02) | 0.0467 | 7/9 | 13.56(1.9) | 13.11(2.02) | 0.0038 |
| Haematocrit | 3/0 | 42.13(5.21) | 41.79(6.03) | 0.33 | 7/1 | 41.21(5.41) | 40.13(6.03) | 0.01 |
| Mean corpuscular volume | 3/0 | 91.26(5.96) | 93.11(6.69) | <0.0001 | 7/1 | 89.94(6.30) | 91.39(6.6) | 0.0052 |
| RDW | 3/0 | 13.18(1.60) | 13.91(1.89) | <0.0001 | 7/1 | 13.49(1.73) | 14.18(2.13) | <0.0001 |
| Platelets | 3/0 | 201.5(82.43 | 171.6(64.93) | <0.0001 | 7/1 | 206.3(82.70) | 174.3(72.93) | <0.0001 |
| Leucocytes | 3/0 | 6.71(2.88) | 7.7(6) | 0.0023 | 7/0 | 6.81(3.43) | 7.15(3.57) | 0.24 |
| Limphocytes | 6/2 | 1.18(0.62) | 1.33(4.81) | <0.0001 | 22/6 | 1.22(0.65) | 0.95(0.60) | <0.0001 |
| Neutrophils | 6/2 | 4.92(2.59) | 5.85(3.33) | <0.0001 | 22/6 | 4.86(2.85) | 5.48(3.16) | 0.0098 |
| Basophils | 6/2 | 0.02(0.02) | 0.02(0.04) | 0.86 | 22/6 | 0.02(0.0006) | 0.02(0.00148) | 0.17 |
| Monoytes | 6/2 | 0.54(0.31) | 0.48(0.47) | 0.03 | 22/6 | 0.52(0.28) | 0.50(0.47) | 0.67 |
| Eosinophils | 6/2 | 0.04(0.14) | 0.02(0.04) | <0.0001 | 22/6 | 0.05(0.27) | 0.02(0.03) | 0.0009 |

NSAIDS: non-steroidal anti-inflammatory drugs; MAX: maximun value; MIN: minimun value; SpO2: pulse oximetric saturation; PaO2: partial arterial oxygen concentration; ALT: alanine aminotransferase; LDH: Lactate dehydrogenase; hs-cTnT: high-sensitivity cardiac troponin T ; RDW: Red blood cell distribution width; VMK100%: standard-high-flow-oxygen-facemask with reservoir-bag at least during six hours and need for more intensive therapy afterwards; Optiflow (TM)-high-flow-nasal-cannula; NIMV: nor invasive mechanical ventilation; ICU : intensive care unit.
